# Smart Grain Storage Solution: Integrated Deep Learning Framework for Grain Storage Monitoring and Risk Alert

**DOI:** 10.3390/foods14061024

**Published:** 2025-03-18

**Authors:** Xinze Li, Wenfu Wu, Hongpeng Guo, Yunshandan Wu, Shuyao Li, Wenyue Wang, Yanhui Lu

**Affiliations:** 1 College of Biological and Agricultural Engineering, Jilin University, Changchun 130022, China; xzli23@mails.jlu.edu.cn (X.L.);; 2 Institute of XinJiang Grain and Oil Science, Urumqi 830000, China; 3 College of Automotive Engineering, Jilin University, Changchun 130022, China

**Keywords:** food security, grain storage, deep learning, multi-model fusion, state monitoring, risk alert

## Abstract

In order to overcome the notable limitations of current methods for monitoring grain storage states, particularly in the early warning of potential risks and the analysis of the spatial distribution of grain temperatures within the granary, this study proposes a multi-model fusion approach based on a deep learning framework for grain storage state monitoring and risk alert. This approach combines two advanced three-dimensional deep learning models, a grain storage state classification model based on 3D DenseNet and a temperature field prediction model based on 3DCNN-LSTM. First, the grain storage state classification model based on 3D DenseNet efficiently extracts features from three-dimensional grain temperature data to achieve the accurate classification of storage states. Second, the temperature prediction model based on 3DCNN-LSTM incorporates historical grain temperature and absolute water potential data to precisely predict the dynamic changes in the granary’s temperature field. Finally, the grain temperature prediction results are input into the 3D DenseNet to provide early warnings for potential condensation and mildew risks within the grain pile. Comparative experiments with multiple baseline models show that the 3D DenseNet model achieves an accuracy of 97.38% in the grain storage state classification task, significantly outperforming other models. The 3DCNN-LSTM model shows high prediction accuracy in temperature forecasting, with MAE of 0.24 °C and RMSE of 0.28 °C. Furthermore, in potential risk alert experiments, the model effectively captures the temperature trend in the grain storage environment and provides early warnings, particularly for mildew and condensation risks, demonstrating the potential of this method for grain storage safety monitoring and risk alerting. This study provides a smart grain storage solution which contributes to ensuring food safety and enhancing the efficiency of grain storage management.

## 1. Introduction

Food is a fundamental human necessity, and food security plays a crucial role in ensuring a nation’s economic development and social stability. As a basic staple, grain not only directly affects the sustenance of the population but also constitutes a vital component of the national socioeconomic framework [1,2,3]. With the continuous growth of the global population and the intensification of climate change, the challenges to food security have become increasingly severe. In this context, the safety of grain storage is critical for maintaining food security. The integrity of grain storage directly impacts both the quality and quantity of stored grain, thereby influencing food market stability and the sustainability of food supply chains [4]. However, inadequate monitoring technologies during the storage process can lead to issues, such as pest infestation, heat buildup, mold growth, and condensation, ultimately resulting in reduced grain quality and large-scale losses [5,6,7,8]. Therefore, effectively monitoring storage states and predicting potential risks within grain piles to ensure the safety of stored grain have become critical issues in contemporary grain security strategies.

In China, most granaries have implemented temperature monitoring systems that utilize temperature-sensing cables within grain piles for the real-time acquisition of temperature data. However, these systems typically only allow the viewing of temperature data in tabular form or the generation of temperature field cloud maps. The assessment of current storage states still heavily relies on the manual expertise of granary managers interpreting these temperature maps, which not only lack real-time responsiveness but also require considerable human effort for data analysis and condition assessment. Consequently, abnormal conditions, such as changes in grain storage quantity, condensation, and mildew, may remain undetected and unresolved in a timely manner. Additionally, existing methods predominantly focus on analyzing current temperature data, without the capability for proactive risk prediction.

The grain storage supervision process primarily involves three key tasks: first, real-time monitoring and assessment of storage states to prevent losses due to spoilage; second, monitoring grain quantities to detect anomalies and prevent theft or mismanagement; and third, anticipating potential risks within grain piles to implement preventive measures and avoid grain loss. In recent years, extensive research by both domestic and international scholars has led to substantial advancements in grain storage supervision.

In the field of quantity monitoring, Zhao et al. proposed a wheat inventory monitoring method based on a Support Vector Regression (SVR) prediction model [9]. By combining multiple factors, such as storage time, weight, and moisture content, and optimizing the kernel function and parameters, they successfully achieved accurate predictions of wheat inventory. Lei et al. proposed a computer vision-based method that utilizes cameras and a two-level spatially constrained Support Vector Machine (SVM) classifier to automatically monitor changes in the grain quantities within granaries [10]. This method can detect real-time changes in the distance between the grain surface and the loading line, thereby assessing the reduction in grain quantity. Duysak et al. developed a grain quantity measurement system using wide beam-width electromagnetic illumination and a scaling model for level measurement in grain silos [11]. By collecting radar backscattering data for various grain amounts and extracting eight features, they utilized the K-nearest neighbor (KNN) algorithm to successfully detect the amount of grain. Enes proposed a 3D level measurement method based on wide beam-width radar that accurately obtains backscatter information from the surface of particles using Compressed Sensing (CS) technology [12]. This method identifies and calculates the 3D coordinates of strong scatter points on the particle surface, ultimately deriving a volume estimation formula. Field tests confirmed the high accuracy of this method using real silo data, effectively addressing the limitations of traditional methods in 3D volume measurement.

In terms of storage state monitoring and assessment, Wu et al. introduced a multi-field coupling theory for grain storage, experimentally validating the interactions among temperature, humidity, and biological factors within grain stacks [13]. This theory facilitates the comprehensive and precise detection of the grain storage state. However, in practical granary applications, the real-time monitoring of parameters, such as grain moisture and internal humidity, within grain piles is hindered by expensive and unreliable equipment, severely limiting the practical implementation of this approach. Currently, most granaries have implemented real-time temperature monitoring with high stability and accuracy through commercially available and established grain temperature monitoring systems. Leveraging the extensive grain temperature data accumulated during long-term storage, Wu et al. utilized the spatiotemporal distribution characteristics of granary temperature fields to assess storage conditions, identifying states such as empty storage, new grain addition, aeration, and risks of condensation and mold growth [14]. These studies effectively demonstrate the feasibility of monitoring storage states using the distribution of temperature fields within grain piles. However, these methods present significant limitations. First, the assessment of storage states based on granary temperature field cloud maps requires the review of all sectional cloud maps to derive a final evaluation, which can result in overlooking the three-dimensional spatial relationships between different points in the grain pile. More importantly, these methods are limited to real-time monitoring of the current storage state and lack the capability to detect potential risks within the grain pile. This shortcoming prevents the prediction of future anomalies or hazards, such as the risk of condensation or mildew. Given the critical importance of early intervention in preventing such risks, to ensure the safety of stored grain and minimize potential losses, the development of methods that integrate risk prediction with storage state monitoring is essential.

In the field of risk prediction, Ge et al. attempted to use convolutional neural network (CNN) to predict the point temperature of grain piles [15]. Li et al. developed an improved SVR model for grain temperature prediction, incorporating external meteorological data to accurately forecast temperature trends within various storage layers [16]. However, these methods predict only average temperatures, limiting their ability to predict localized temperature anomalies and associated storage risks within grain piles. Yin et al. constructed models for grain temperature and vapor pressure fields, simulating condensation processes under different temperature gradients to provide quantitative predictions of localized condensation events [17]. Nevertheless, acquiring real-time moisture and vapor pressure data within grain stacks remains a challenge in practical storage settings. Wang et al. analyzed the relationship between storage mold growth and temperature-humidity fields using grain temperature and humidity field cloud maps [18]. However, these methods suffer from poor real-time detection capabilities and the difficulty of obtaining accurate humidity data. Without rapid diagnosis and precise interventions, spoilage within grain piles can escalate quickly, leading to severe storage safety issues. In order to provide a grain storage monitoring method that can simultaneously monitor the current storage status in real-time and predict potential risks within the grain pile, this paper proposes an integrated deep learning framework for grain storage monitoring and risk alert. By integrating a 3D DenseNet model with a 3D Convolutional Neural Network—Long Short-Term Memory (3DCNN-LSTM) model, the proposed method enables the accurate assessment of the current storage state and prediction of potential risks within grain piles. Here 3D DenseNet is employed to classify the current storage state and quantity based on three-dimensional grain temperature data. Historical grain temperature data are then input into the 3DCNN-LSTM model to forecast future temperature fields, which are subsequently analyzed by 3D DenseNet to predict potential risks, including the location and timing of anomalies, such as condensation and mildew. This integrated approach allows for the precise real-time assessment of storage states and proactive risk prediction, enabling granary managers to implement timely interventions to prevent grain loss and ensure grain security.

## 2. Materials and Methods

### 2.1. Data Sources

**Figure 1:**
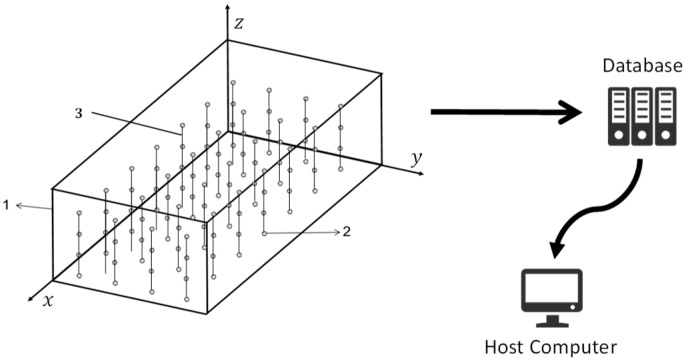
Example of the grain temperature monitoring system. 1. Granary; 2. temperature sensor; and 3. cable.

Currently, most granaries in China have implemented real-time temperature monitoring systems for grain storage, as illustrated in Figure 1. Temperature-sensing cables are arranged within the grain pile, with M × N × H temperature sensors placed along the *X*, *Y*, and *Z* axes to achieve real-time grain temperature detection. Since the grain temperature monitoring systems installed in different granaries are sourced from various manufacturers, the temperature sensors used are also diverse, with many systems utilizing the DS18B20 digital temperature sensor. The collected grain temperature data are then transmitted to a local or cloud database via either wired or wireless communication. The database stores daily grain temperature data, including the detection time and the three-dimensional coordinates of the sensors in the granary. The daily grain temperature data for each granary form a 3D matrix to describe the temperature field distribution within the grain pile. The 3D coordinates of the grain temperature data are represented by the sensor identifiers in the *X*, *Y*, and *Z* directions. The moisture data of the stored grain, typically including measurements obtained through drying methods and regularly sampled moisture data, are generally detected by the granary staff and manually uploaded to the database via the host computer. Furthermore, the host computer enables the viewing and downloading of historical grain temperature and moisture data. The dataset used in this study includes publicly available datasets published by our team [14], comprising 186,611 data samples collected from 437 flat granaries in 10 different provinces of China, from 1 June 2017 to 1 December 2018. The grain varieties included in the dataset are wheat, corn, and rice. Additionally, to increase the diversity of the dataset and improve the model’s generalization and robustness, temperature data from 82 flat granaries in Xinjiang and Jilin provinces were collected between January 2023 and June 2024, forming a new dataset.

### 2.2. Data Preprocessing

During the collection of grain temperature data, issues such as sensor damage and missing data uploads may arise, necessitating data preprocessing. Initially, data are cleaned using reasonable threshold values to remove common erroneous data from the grain temperature control system, such as 888 °C, −85 °C, and 85 °C. Furthermore, Z-scores are used to remove outliers from the grain temperature data. First, the mean μ and standard deviation σ of the grain temperature data are calculated. Then, the *Z*-score for each data point is computed using Formula (1), and any data point with an absolute *Z*-Score greater than 3 is considered an outlier and removed. The missing values are filled using mean interpolation based on the averages of adjacent data points in the time series.

(1) $$Z=\frac{X-\mu }{\sigma }$$

where *X* represents the value of each data point.

After data preprocessing, a detailed statistical analysis of the dataset was conducted to support subsequent model training and evaluation. The samples were classified according to different storage states for training the 3D DenseNet-based storage state classification model. The storage state labels in the dataset include six states: normal storage, empty storage, aeration, new grain addition, condensation, and mildew. Each state corresponds to distinct grain temperature variation characteristics, making accurate classification crucial for model training. Empty storage, aeration, and new grain addition are identified through operation logs and inventory records from each granary. For each granary identified as having a mildew or condensation state based on temperature data analysis, approximately 1 m^3^ of grain samples was collected using a mechanical sampler to verify the judgment results. The specific classification details of the dataset are presented in Table 1. Since the temperature sensor layouts within grain piles vary across different granaries, all samples were resized to ensure spatial consistency, thus facilitating subsequent model input processing. This study used spline interpolation to resize all 3D matrices to a unified 10 × 6 × 4 dimension [14]. Spline interpolation is a method used to construct a smooth curve through a set of data points. In this context, it is employed to resize the 3D matrices by fitting a piecewise polynomial function to the temperature data, thereby preserving the spatial structure while achieving the desired dimensions. This process not only ensures data consistency but also maximizes the preservation of the original spatial features of the data. After resizing, the data were standardized.

### 2.3. Construction of Grain Storage State Classification Model Dataset

In this study, to address the class imbalance issue in the dataset, the sample size of abnormal storage samples was expanded by flipping the 3D grain temperature matrices along the *X*, *Y*, and *Z* axes, thereby quadrupling the sample size. Additionally, data augmentation was performed using Generative Adversarial Networks (GANs). GANs, through an adversarial training mechanism, can generate synthetic samples that are highly consistent with the real data distribution. They are commonly used to address the class imbalance problem in deep learning classification tasks by effectively increasing the sample size of the minority class, thereby improving the generalization and robustness of the classification model [19,20]. All 3D grain temperature matrices corresponding to the condensation and mildew categories were extracted from the original dataset and utilized as real samples to train the GAN generator.

GAN consists of two main components: the generator and the discriminator. The generator converts random noise vectors into realistic 3D grain temperature matrices. It consists of multiple deep convolutional and upsampling layers, progressively constructing the spatial structure of the 3D data. The generator receives a 100-dimensional random noise vector, which is mapped through a fully connected layer to a lower-resolution 3D feature map. After a series of fully connected and transpose convolution layers, the spatial resolution is gradually enhanced through three transpose convolutions and upsampling operations, ultimately generating a 3D grain temperature matrix that matches the real data structure. The discriminator is responsible for distinguishing whether the input 3D grain temperature matrix is real data or synthetic data generated by the generator. The discriminator consists of two 3D convolutional layers, which progressively extract spatial features from the data. Finally, a fully connected layer outputs a probability value, indicating the likelihood that the input data is real. To enhance the robustness of the discriminator, ReLU activation functions and Dropout regularization layers were introduced to prevent overfitting. Ultimately, the discriminator outputs a probability value through a Sigmoid activation function to assess the authenticity of the input data. By rotating the samples and using GANs for data augmentation, the sample sizes of the condensation and mildew categories were increased to 980 and 360, respectively. Considering that the sample size of the normal storage category far exceeds that of the other categories, some normal storage samples were removed to reduce its sample size and make the dataset more balanced. The dataset for grain storage state classification was constructed with 6000 normal storage samples, 2148 empty storage samples, 1248 aeration samples, 908 new grain addition samples, 980 condensation samples, and 360 mildew samples.

### 2.4. Construction of Grain Temperature Prediction Model Dataset

**Figure 2:**
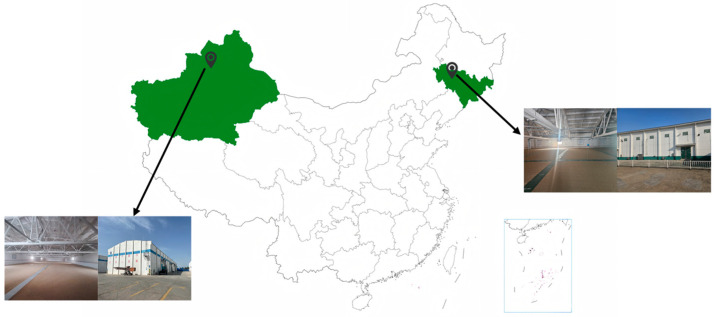
Source of data set of grain temperature prediction model.

Six granaries with complete data on daily grain temperature and grain moisture content from January 2023 to June 2024 were selected to construct the dataset for the grain temperature prediction model. Three granaries are located in Xinjiang, China, storing wheat, while the other three granaries are located in Jilin Province, storing corn. All granaries are flat warehouses, as shown in Figure 2. Statistical descriptions of the grain temperature data in the dataset were performed to gain a deeper understanding of the data’s distribution characteristics and temporal dependencies. The mean, median, standard deviation, maximum, minimum, skewness, and kurtosis of the grain temperature data were calculated, with the results shown in Table 2. The mean grain temperature is 7.27 °C, and the median is 6.06 °C. Their proximity indicates a relatively balanced distribution, with no significant skew. The standard deviation is 11.41 °C, indicating a large range of fluctuations and significant temperature variability. The maximum temperature is −15.7 °C and the minimum temperature is 38.7 °C, suggesting the presence of extreme temperature values, likely caused by abrupt changes in the external environment. The skewness is 0.17, close to zero, indicating that the temperature data distribution is nearly symmetric, with no significant skew. The kurtosis is −0.98, indicating a relatively flat distribution with a low frequency of extreme values, and relatively stable temperature fluctuations [21].

**Figure 3:**
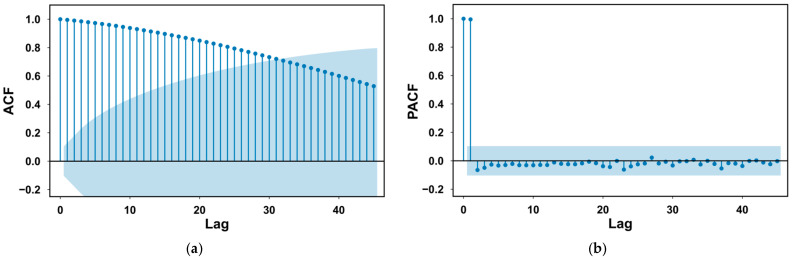

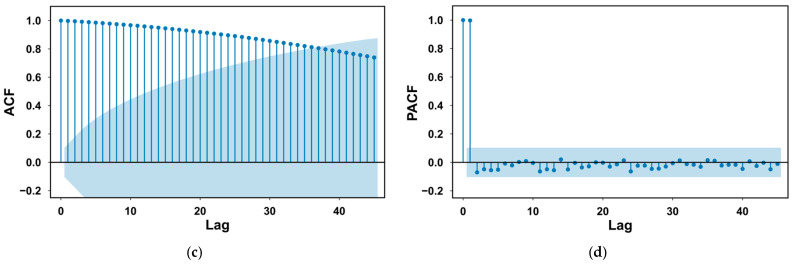
Correlation analysis of grain temperature. (**a**) ACF diagram of granary 1; (**b**) PACF map of granary 1; (**c**) ACF diagram for granary 2; and (**d**) PACF map of granary 2.

To further explore the time-series characteristics of the data, one granary from each of the two regions in the grain temperature prediction dataset was selected, and one year of historical grain temperature data was analyzed. Daily average temperatures were calculated, followed by analysis using the Autocorrelation Function (ACF) and Partial Autocorrelation Function (PACF) [22]. The ACF and PACF plots were computed and drawn, as shown in Figure 3. The ACF plot shows significant autocorrelation that persists up to a lag of 30–35 days, indicating that data from the past 30 to 35 days still have a significant effect on the current values. The PACF truncates after lag 2, indicating that the primary direct correlation is concentrated within the first 2 days. Based on the results of the ACF and PACF analysis, we initially selected a time window of 30 to 40 days when constructing the 3DCNN-LSTM-based grain temperature prediction model, to fully utilize seasonal information.

### 2.5. Integrated Deep Learning Framework for Grain Storage Monitoring and Risk Alert

#### 2.5.1. Algorithm Architecture

The proposed grain storage state monitoring and risk alert method based on a deep learning framework consists of two main components: (1) a grain storage state classification model based on 3D DenseNet; and (2) a grain temperature prediction model based on 3DCNN-LSTM. First, current and historical grain temperature data from the granary are collected, and the current grain temperature data are processed into a standardized 10 × 6 × 4-sized 3D matrix, which is then fed into the 3D DenseNet grain storage state classification model. The model classifies the current storage state as one of the following: normal storage, empty storage, aeration, new grain addition, condensation, or mildew. Historical grain temperature data are input into the 3DCNN-LSTM model for the grain temperature prediction task. The 3DCNN module captures the spatial dependencies between temperature points, and its output is then used as input to the LSTM module. The LSTM module performs time-series forecasting to generate predicted results for the grain temperature field, which are subsequently mapped back to the original temperature data space by the output layer to produce the final grain temperature prediction. The predicted grain temperature is subsequently fed into the 3D DenseNet-based grain storage state detection model to predict the location and timing of potential condensation and mildew risks. Condensation within grain piles occurs when water vapor in the air cools down and condenses into liquid droplets, which then adhere to the grain or the walls of the granary. This phenomenon typically arises in environments with a significant temperature gradient within the grain pile or high humidity. Condensation results in the formation of water droplets on the surface of the grain, thereby increasing the risk of fungal growth and potentially leading to the accumulation of vapor plaques. Mildew usually occurs in damp environments with high temperatures and excessive moisture in the grain, conditions that promote fungal growth. The mold that develops on the grain surface can cause spoilage and lead to a decline in grain quality. Currently, most granaries lack real-time, accurate monitoring systems for critical parameters, such as moisture content, humidity, carbon dioxide levels, and microbial activity, within the grain pile. Consequently, it is challenging to directly confirm the occurrence of condensation and mold growth through storage state monitoring alone. The approach proposed in this paper relies on monitoring grain temperature and absolute water potential to predict regions within the grain pile that are at higher risk of condensation or mold formation. This predictive assessment, referred to as “condensation and mildew risks”, assists in identifying areas more likely to encounter these challenges. However, confirming the actual presence of condensation and mold still requires physical sampling within the grain pile. Despite this limitation, the risk prediction capability offers a valuable early warning system, facilitating timely preventive measures to ensure the long-term safety and quality of stored grain.

#### 2.5.2. Grain Storage State Classification Model Based on 3D DenseNet

**Figure 4:**
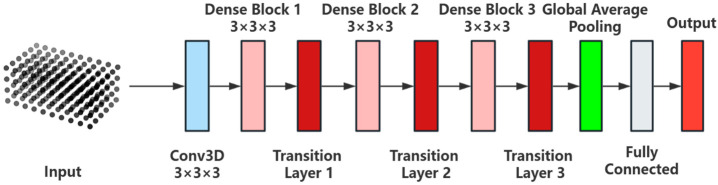
Model structure of 3D DenseNet.

In this study, a grain storage state classification model based on the 3D DenseNet architecture is proposed, aimed at effectively identifying various storage states, including normal storage, empty storage, aeration, new grain addition, condensation, and mildew; 3D DenseNet is a three-dimensional extension of the DenseNet architecture, which employs a dense connection design philosophy that links the output of each layer to the outputs of previous layers, significantly improving feature reuse and propagation efficiency [23]. This design facilitates the flow of information within deep networks and enables the efficient capture of spatial features for the grain storage state classification task. The model structure is shown in Figure 4. The input to the model is a 10 × 6 × 4-sized 3D grain temperature matrix, which represents the spatial and temperature distribution of the grain storage state. First, feature extraction is performed using an initial 3D convolutional layer. The convolutional layer uses a 3 × 3 × 3 kernel to effectively capture the local spatial features of the input data and generate feature maps.

The core structure of the 3D DenseNet model consists of multiple Dense Blocks, each performing feature extraction through several convolutional layers and utilizing dense connections to merge the output of each layer with the outputs of all preceding layers. This structure enables the model to fully leverage the feature information from each layer, enhancing the diversity of feature representation and the ability to capture detailed patterns. To prevent excessive expansion of feature dimensions and reduce computational burden, a Transition Layer is introduced after each Dense Block. The Transition Layer compresses the number of channels through convolution operations and reduces the spatial dimensions using average pooling. This not only alleviates the computational burden but also helps the model focus on more important features, thereby preventing overfitting. After feature extraction and compression, the model uses a Global Average Pooling layer to compress the multi-dimensional feature maps into a one-dimensional vector. This vector is passed into a fully connected layer, which outputs the probability distribution of the six grain storage states through a Softmax activation function, completing the multi-class classification task. The advantage of the 3D DenseNet architecture lies in its dense connection strategy, where the features of each layer are retained and shared with subsequent layers, enabling the network to effectively propagate gradients through deeper structures, alleviating the gradient vanishing problem in traditional deep networks. This architecture not only improves the model’s training efficiency and stability but also enhances the model’s robustness in small sample categories through efficient feature sharing. This design demonstrates strong classification performance in grain storage state classification tasks, especially in cases of imbalanced data categories, where the model’s accuracy and adaptability are significantly improved.

#### 2.5.3. Grain Temperature Prediction Model Based on 3DCNN-LSTM

In this study, a hybrid model based on a 3D Convolutional Neural Network (3D-CNN) and Long Short-Term Memory (LSTM) network is designed for the accurate prediction of grain temperature changes. The 3D-CNN module is primarily responsible for capturing spatial dependencies in 3D data by using multiple 3D convolutional and pooling layers to extract spatial features of grain temperature in depth, height, and width directions, fully utilizing spatial information. The LSTM module captures temporal dependencies in the grain temperature data by processing time-series data and learning the dynamic patterns of grain temperature changes over time. By combining the 3D-CNN and LSTM, the model can simultaneously capture the spatial and temporal dependencies of grain temperature data. Additionally, because the temperature field within the grain pile is influenced by various factors, such as temperature, humidity, moisture, and microorganisms [13,24], relying solely on historical grain temperature data for future predictions may lead to insufficient accuracy. Considering that most granaries in China currently have the capacity for real-time temperature monitoring and periodic moisture sampling, this study introduces the variable of absolute water potential as a supplementary input to the model. This variable, along with historical grain temperature data, is utilized to predict grain temperature. Absolute water potential reflects the freedom and energy state of moisture within the grain and is closely related to factors such as grain moisture content, variety, and temperature. These factors collectively influence the heat and mass transfer behavior within the grain pile. Its calculation follows the Formula (2) proposed in reference [25], which provides a comprehensive representation of the grain storage conditions in the granary. This approach not only compensates for the limitations of relying on temperature data alone to reflect the overall storage condition but also enhances the model’s understanding and predictive capability regarding the granary environment, all while maintaining computational efficiency.

(2) $$E_{jg}=8.31\times (T_{g}+273)\times \ln(\exp(\frac{(\frac{D}{222}\times (e^{\frac{B1-M}{A1}}-e^{\frac{B2-M}{A2}})+0.9854)\times (1737.1-\frac{474242}{273+T_{g}})+D\times (1-e^{\frac{B1-M}{A1}})-68.57}{87.72})\times 133.3)/18$$

where *E_jg_* represents absolute water potential of grain (KJ/Kg), *T_g_* represents grain temperature (°C), *M* represents grain moisture content (%), *A*1, *A*2, *B*1, *B*2, *D* represent fitting coefficients of different grain varieties [25].

**Figure 5:**
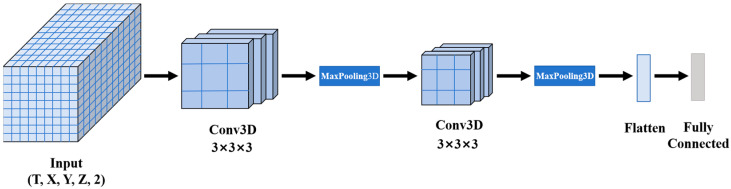
Module structure of 3DCNN.

The 3D Convolutional Neural Network is an extension of the traditional 2D Convolutional Neural Network model, specifically designed for handling 3D data [26]. It captures local spatial features of the input data by sliding a convolutional kernel over the three-dimensional space (*X*, *Y*, *Z*). The 3D-CNN model is capable of effectively capturing complex patterns in spatial dimensions, offering significant advantages when analyzing time-series data with a three-dimensional structure. In the grain temperature field prediction task, the 3D-CNN module captures spatial features through layer-by-layer convolution and pooling operations, providing rich input features for subsequent time-series prediction. The 3DCNN module in the model consists of five main parts: the input layer, convolutional layer, pooling layer, Flatten layer, and fully connected layer, as shown in Figure 5.

The input layer is primarily responsible for receiving the preprocessed grain temperature data from different location points within the granary, organizing it into a four-dimensional tensor with the shape (*T*, *X*, *Y*, *Z*, 2). *T* represents the number of time steps; *X*, *Y*, and *Z* are the coordinates of the sensor location points; and 2 represents dual channels: grain temperature and absolute water potential. Since both the grain temperature data and absolute water potential data are three-dimensional in space, 3D convolution is used as the convolution function, enabling the simultaneous capture of spatial features across the length, width, and height dimensions. The 3D convolution operation slides a 3D convolutional kernel over the three spatial dimensions (*X*, *Y*, *Z*) of the input data, extracting features from local regions. In the convolution operation, for the input tensor *I* and convolutional kernel *W*, the value at position (*i*, *j*, *k*) in the output feature map *O* is calculated as:

(3) $$O(i,j,k)=\sum_{m=0}^{KX-1}\sum_{n=0}^{KY-1}\sum_{p=0}^{KZ-1}W(m,n,p)\times I(i+m,j+n,k+p)+b$$

where *W*(*m*, *n*, *p*) represents the weight at position (*m*, *n*, *p*) in the convolutional kernel, *I*(*i* + *m*, *j* + *n*, *k* + *p*) represents the corresponding element value in the input tensor corresponding to the convolutional kernel, *b* represents the bias term, which adjusts the output of the convolution operation.

The pooling layer serves to downsample the output of the convolutional layer, reducing the spatial dimensions of the data and thereby lowering computational complexity, while preserving the primary spatial features. Max-Pooling is selected as the pooling method, which takes the maximum value within each local region. Max-Pooling retains the most significant features while reducing the size of the feature map, thus mitigating the risk of overfitting. The Flatten layer flattens the three-dimensional output feature map from the pooling layer into a one-dimensional vector, which is then passed into the fully connected layer for final feature fusion and output before being input into the LSTM module.

**Figure 6:**
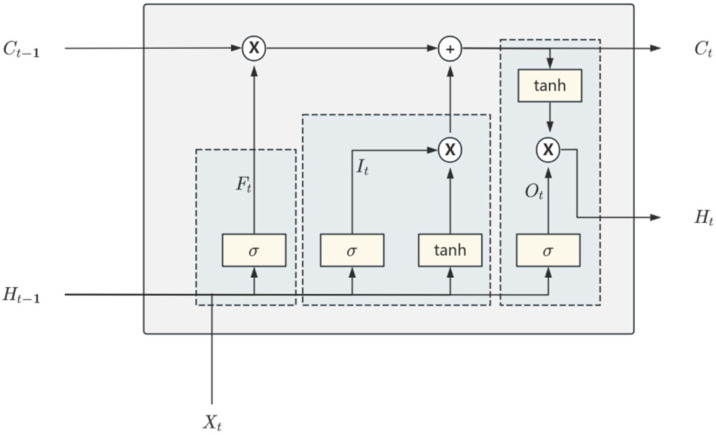
LSTM module structure.

LSTM is a specialized type of Recurrent Neural Network (RNN) designed to address the vanishing or exploding gradient problem encountered by traditional RNNs when processing long sequences during backpropagation [27]. The LSTM alleviates this problem effectively by introducing gating mechanisms and memory units. In the 3DCNN-LSTM grain temperature prediction model, the LSTM module receives the feature vectors output by the 3D-CNN module as time-series data. The feature vector *X_t_* for each time step is input into the LSTM unit for time dependency modeling, where the LSTM learns the dynamic patterns of grain temperature changes over time through its internal gating mechanisms. The internal structure of the LSTM module is shown in Figure 6. For each time step *t*, the LSTM receives the current input vector *X_t_* and maintains the previous hidden state *H*_*t*__−__1_ and the previous cell state *C*_*t*__−__1_. The forget gate *F_t_* first controls which information in the cell state should be forgotten, with the computation process shown in Equation (4). The input gate adjusts how much of the current input information is written into the cell state, shown in Equation (5). The candidate cell state generates new information, which is prepared to be added to the cell state, shown in Equation (6). The cell state Ct is updated, integrating the outputs of the forget and input gates, computed in Equation (7). The output gate *O_t_*, through a linear transformation and Sigmoid activation function, decides which parts of the cell state will be output, mathematically expressed in Equation (8), controlling the impact of the cell state on the hidden state output.

(4) $$F_{t}=\sigma (W_{f}X_{t}+U_{f}H_{t-1}+b_{f})$$

(5) $$I_{t}=\sigma (W_{i}X_{t}+U_{i}H_{t-1}+b_{i})$$

(6) $${\tilde{C}}_{t}=\tanh(W_{c}X_{t}+U_{c}H_{t-1}+b_{c})$$

(7) $$O_{t}=\sigma (W_{o}X_{t}+U_{o}H_{t-1}+b_{o})$$

(8) $$C_{t}=F_{t}\times C_{t-1}^{*}+I_{t}\times {\tilde{C}}_{t}$$

where, *X_t_* represents the current input, *H_t−_*_1_ represents the previous hidden state, {*W_f_*, *U_f_*, *b_f_*}, {*W_i_*, *U_i_*, *b_i_*}, {*W_o_*, *U_o_*, *b_o_*}, {*W_c_*, *U_c_*, *b_c_*} are the learned weight coefficients of the gating mechanisms during the model training process, and σ represents the Sigmoid function, applied to the gating mechanisms.

The hidden state is updated to *H_t_*, mathematically expressed by Equation (9). These gating mechanisms work together, enabling LSTM to effectively capture both short-term and long-term dependencies in sequence data, selectively remembering and forgetting information, thereby enhancing the model’s ability to model sequential data. Finally, the hidden state of LSTM, *H_t_* is mapped to the predicted 3D grain temperature matrix through a fully connected layer, generating predicted temperature values for multiple spatial points, forming a 3D grain temperature prediction matrix.

(9) $$H_{t}=O_{t}\times \tanh(C_{t})$$

### 2.6. Model Training and Hyperparameter Tuning

The model training and hyperparameter tuning in this study were conducted on a T7920 graphics workstation. The workstation is configured with dual 4214R processors and an RTX A4000-16G GPU. The grain storage state classification model dataset was randomly divided into training, validation, and test sets in a 70%, 15%, and 15% ratio, respectively. The grain temperature prediction model dataset was divided in chronological order into training, validation, and test sets in a 70%, 15%, and 15% ratio, respectively. Both models employ the Adam optimizer, an adaptive optimization algorithm that adjusts the learning rate for each parameter based on the first and second moments of the gradients. This self-adjusting mechanism facilitates faster and more efficient convergence, particularly in the presence of noisy or sparse gradients, and minimizes the need for fine-tuning hyperparameters. Early stopping is also incorporated to prevent overfitting. The grain storage state classification model employs a Weighted Cross-Entropy loss function, which dynamically adjusts the class weights based on the sample size of each class to ensure that the model focuses more on minority class samples during training. The grain temperature prediction model utilizes a sliding time window for training, converting time-series data into fixed-length input sequences with a target prediction period of 10 days. The Grid Search method is employed for systematic hyperparameter tuning [28]. The specific hyperparameter ranges and optimal settings for both models are presented in Table 3 and Table 4.

### 2.7. Evaluation Metrics

For the grain storage state classification model, performance is evaluated using classification metrics such as precision, accuracy, macro-average F1 score, recall, and the confusion matrix [29].

Precision: represents the proportion of correctly classified samples among all samples classified by the algorithm.

(10) $$\mathrm{Pr}ecision=\frac{TP}{TP\cup FP}$$

Recall: represents the proportion of correctly classified samples among those manually labeled as needing classification.

(11) Recall=TPTP∪FN

F1 score: is used to measure the combined performance of precision and recall and is particularly suitable for situations where the sample sizes of categories in the dataset are imbalanced.

(12) $$F1=\frac{\;2\;\times \;\mathrm{Precision}\;\times \;\mathrm{Recall}}{\mathrm{Rrecision}+\mathrm{Recall}}$$

Accuracy: represents the proportion of correctly classified samples across all categories relative to the total number of observations.

(13) $$Accuracy=\frac{TP+TN}{TP+TN+FP+FN}$$

In these formulas, *TP* (True Positive) represents the number of samples that are true positives and predicted as positives; *TN* (True Negative) represents the number of samples that are true negatives and predicted as negatives; *FP* (False Positive) represents the number of samples that are true negatives but predicted as positives; *FN* (False Negative) represents the number of samples that are true positives but predicted as negatives.

Confusion matrix: The confusion matrix is a tool used to evaluate the performance of a classification model, providing the classification accuracy and error distribution for each class by displaying the relationship between the true and predicted categories. Introducing the confusion matrix allows for an intuitive understanding of the model’s performance across different categories, thereby providing a comprehensive evaluation of the multi-class classification model’s ability.

For the grain temperature prediction model, performance is evaluated using RMSE (Root Mean Squared Error) and MAE (Mean Absolute Error) [30]. The formulas are as follows:

(14) $$MAE=\frac{1}{K}\sum_{t=1}^{K}\left|{\hat{Y}}_{t}-Y_{t}\right|$$

(15) $$RMSE=\sqrt{\frac{\sum_{t=1}^{K}({\hat{Y}}_{t}-Y_{t}{)}^{2}}{K}}$$

where *K* represents the number of output samples, *Y_t_* represents the actual grain temperature value, and ${\hat{Y}}_{t}$ represents the predicted grain temperature value.

## 3. Results and Discussion

### 3.1. Comparison of Grain Storage State Classification Model Performance

To comprehensively evaluate the performance of the proposed 3D DenseNet-based grain storage state classification model, comparison experiments were conducted with several deep learning models, which have demonstrated strong performance in 3D data classification tasks, selected as baseline models. The 3D EfficientNet model: EfficientNet, through a compound scaling strategy, can scale the depth, width, and resolution of the network while maintaining efficient computation, allowing it to provide high classification accuracy even with limited computational resources [31]. The 3D ResNet model: 3D ResNet extends the classic residual network structure into three-dimensional space, using residual connections to address the gradient vanishing problem in deep networks, and it possesses strong feature representation and generalization capabilities [29]. The 3D InceptionV3 model: 3D InceptionV3 extracts spatial features using multi-scale convolutional kernels, enabling it to capture spatial information at different scales, making it suitable for handling data with complex spatial variation patterns [32]. After training all four models on the same dataset, classification was performed on the test set samples, and evaluation metrics were calculated for model comparison, as shown in Table 5.

Table 5 lists the main evaluation metrics for each model in the grain storage state classification task, including accuracy, precision, recall, and F1 score. The 3D DenseNet module performed best across all metrics, achieving an accuracy of 97.38%, precision of 0.9596, recall of 0.9602, and an F1 score of 0.9585. This exceptional classification performance indicated that 3D DenseNet could efficiently extract and reinforce key features, providing significant advantages when handling imbalanced 3D grain temperature matrix data. In comparison, the 3D ResNet model achieved an accuracy of 96.62%, precision of 0.9506, recall of 0.9431, and an F1 score of 0.9467, which, although slightly lower than 3D DenseNet, still demonstrated high classification performance. The performances of the 3D InceptionV3 and 3D EfficientNet models were relatively weaker, with accuracy rates of 94.93% and 92.72%, and F1 scores of 0.9135 and 0.8790, respectively. These results indicate that 3D DenseNet provides high-precision classification capabilities for the grain storage state classification task, offering reliable technical support for state monitoring and decision-making in practical grain storage management.

**Figure 7:**
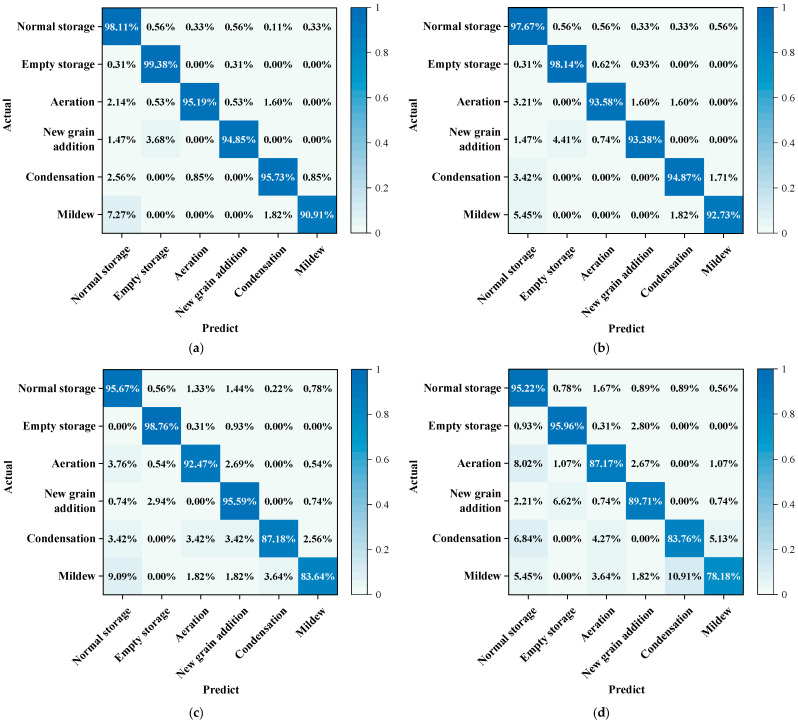
Confusion matrix for grain storage states classification of different models. (**a**) 3D DenseNet; (**b**) 3D ResNet; (**c**) 3D InceptionV3; and (**d**) 3D EfficientNet.

A further comparison of model performance across specific categories is presented in Figure 7, which shows the classification confusion matrices for 3D DenseNet (Figure 7a), 3D ResNet (Figure 7b), 3D InceptionV3 (Figure 7c), and 3D EfficientNet (Figure 7d). For the categories of normal storage, empty storage, and aeration, 3D DenseNet performed the best, achieving accuracy rates of 98.11%, 99.38%, and 95.19%, respectively, demonstrating strong classification ability. The accuracy rates of 3D ResNet are 97.67%, 98.14%, and 93.58%, which are close but slightly lower than those of 3D DenseNet. In contrast, 3D InceptionV3 and 3D EfficientNet performed slightly worse in these three categories. For the new grain addition category, 3D DenseNet achieved an accuracy of 94.85%, continuing its strong overall classification performance. The accuracy of 3D ResNet was 93.38%, which, although slightly lower, still demonstrated good performance. Notably, 3D InceptionV3 performed best in the new grain addition category, achieving an accuracy of 95.59%, surpassing the other three models, indicating its advantage in this specific category.

In grain temperature prediction for storage risk alerting, the primary focus is on providing warnings for condensation and mildew risks. Therefore, when comparing the performance of grain storage state classification models, the ability to detect condensation and mildew states is particularly crucial. Furthermore, 3D DenseNet continues to perform excellently in these two minority categories: in condensation, the accuracy is 95.73%, significantly higher than the other models; in mildew, the accuracy is 90.91%, slightly lower than 3D ResNet’s 92.73%, but still demonstrating strong feature extraction and category identification capabilities. Overall, 3D DenseNet performed excellently and consistently across various comparisons. Although 3D ResNet performed slightly better in the mildew category and 3D InceptionV3 had higher accuracy in the new grain addition category, 3D DenseNet remained the strongest model overall, achieving the best balance between classification performance, feature extraction efficiency, and robustness. Based on these results, 3D DenseNet is not only suitable for the daily monitoring of grain storage states but also provides timely and accurate alerts for potential storage risks, such as condensation and mildew, and is supported by the grain temperature prediction model, demonstrating broad practical application prospects.

### 3.2. Comparison of Grain Temperature Prediction Model Performance

To verify the advantages of the proposed 3DCNN-LSTM model in grain temperature field prediction, 3D Convolutional Neural Network—Gated Recurrent Unit (3DCNN-GRU), LSTM, 3DCNN-LSTM-1, and 3DCNN-LSTM were selected for comparative analysis. The 3DCNN-GRU model combines the advantages of the 3D convolutional network for extracting spatial features and Gated Recurrent Unit (GRU) for capturing temporal dependencies, efficiently handling spatial and temporal relationships in spatiotemporal data. LSTM (Long Short-Term Memory) is a model widely used for time-series prediction, effectively capturing long-term dependencies in sequential data. However, LSTM has a relatively weak ability to capture the spatial structure of input data, which may limit its effectiveness when handling tasks with significant spatial features. Using LSTM as the baseline model, the goal is to verify the advantages of the 3D-CNN module in capturing spatial relationships within the grain temperature field and assess its impact on improving grain temperature prediction accuracy. A single-channel 3DCNN-LSTM model (named 3DCNN-LSTM-1) was also designed, which inputs only historical grain temperature data to explore the impact of introducing absolute water potential on the grain temperature prediction accuracy. Comparative analysis with the aforementioned models systematically evaluated the impact of different model architectures on grain temperature prediction accuracy, further validating the advantages of the proposed 3DCNN-LSTM model. The aforementioned models were trained on the same dataset, and after training and hyperparameter tuning, the evaluation metrics of each model were computed on the test set, with the results shown in Table 6.

**Figure 8:**
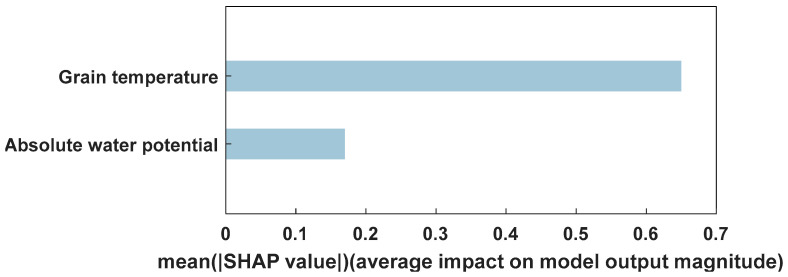
Importance of input variables in 3DCNN-LSTM model.

As shown in the evaluation metric results in Table 6, the proposed 3DCNN-LSTM model outperformed all baseline models on all three evaluation metrics, achieving the lowest MAE (0.24 °C) and RMSE (0.28 °C). Compared to the other three models, the MAE was reduced by 17.24%, 29.41%, and 11.11%, and the RMSE was reduced by 12.5%, 26.31%, and 9.68%, respectively. The model combined 3D Convolutional Neural Networks with LSTM. The 3D convolution module effectively captured the spatial features of the grain temperature field, while LSTM accurately modeled the long-term dependencies in time-series data. This enabled 3DCNN-LSTM to leverage both spatial and temporal information, resulting in superior prediction accuracy compared to other models. In contrast, while 3DCNN-GRU also combined the 3D convolution module to extract spatial features, GRU did not model time dependencies as effectively as LSTM, resulting in slightly lower accuracy. The LSTM model performed well in time-series modeling but lacked spatial feature integration, resulting in lower overall prediction accuracy. Additionally, the 3DCNN-LSTM-1 model used only historical grain temperature data for temperature prediction, without incorporating absolute water potential as an input variable. The MAE of the 3DCNN-LSTM-1 model was 0.27 °C and the RMSE was 0.31 °C, while the MAE and RMSE of the 3DCNN-LSTM model were 0.24 °C and 0.28 °C, respectively. This indicates that the prediction accuracy improved with the inclusion of absolute water potential as an additional input variable, resulting in an 11.1% reduction in MAE and a 9.7% reduction in RMSE. To further validate these results, a sensitivity analysis was conducted using the SHAP method to assess the contribution of grain temperature and absolute water potential to the temperature prediction outcomes. SHAP, a feature importance measurement method based on game theory, is widely used to interpret complex machine learning models. By assigning a contribution value to each input feature, SHAP effectively reveals the model’s dependency on each feature and aids in understanding the prediction mechanism [33]. The SHAP results, presented in Figure 8, show that the average absolute SHAP value for grain temperature is 0.65 °C, while the average absolute SHAP value for absolute water potential is 0.17 °C. The SHAP values clearly indicate the dominant role of grain temperature in the prediction results, while the inclusion of absolute water potential serves an auxiliary role, enhancing the model’s predictive accuracy.

**Figure 9:**
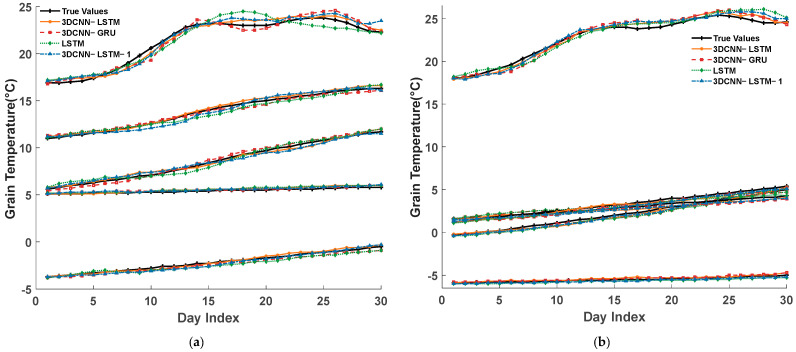
The predicted and true values of grain temperature at each point in the forecast period. (**a**) Jilin No. 3 Granary and (**b**) Xinjiang No. 1 Granary.

**Figure 10:**
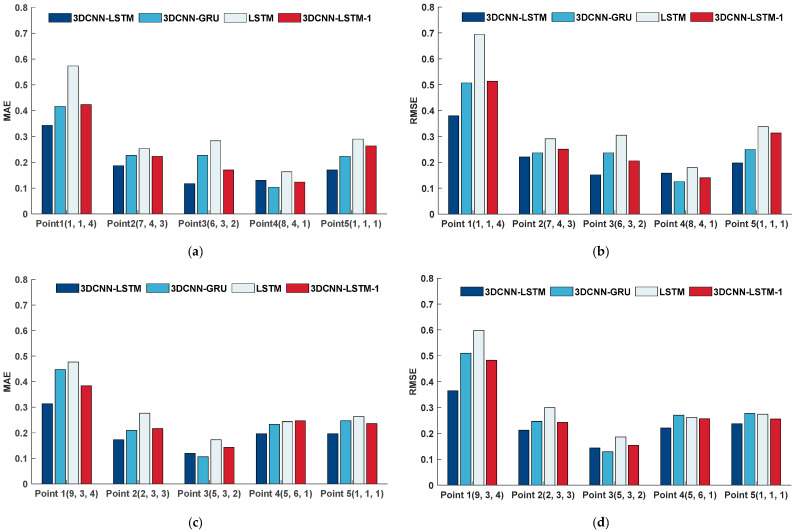
Grain temperature prediction evaluation coefficients at different locations in granary. (**a**) MAE evaluation coefficients for grain temperature prediction at different locations in Jilin No. 3 granary using different models; (**b**) RMSE evaluation coefficients for grain temperature prediction at different locations in Jilin No. 3 granary using different models; (**c**) MAE evaluation coefficients for grain temperature prediction at different locations in Xinjiang No. 1 granary using different models; and (**d**) RMSE evaluation coefficients for grain temperature prediction at different locations in Xinjiang No. 1 granary using different models.

To further compare the prediction performance of the 3DCNN-LSTM model with the other three models at different location points within the granary, one granary was randomly selected from each data source in the grain temperature dataset (Jilin No. 3 granary and Xinjiang No. 1 granary), with one location point randomly selected from each height level along the *Z*-axis of the grain temperature matrix for each granary. Additionally, to account for radial temperature variations within the XY cross-section of the grain pile, one location point at the bottom of the grain pile, closest to the coordinate axis origin (1, 1, 1), was selected. The prediction performance of the four models at each location was compared, and the evaluation metrics were calculated. The predicted and actual grain temperatures at each point for all models in the two granaries over a 30-day period are shown in Figure 9, with the evaluation metric calculation results displayed in Figure 10.

From Figure 9 and Figure 10, it can be seen that the 3DCNN-LSTM model consistently maintains the same stable trend as the actual grain temperatures, demonstrating good prediction accuracy. It effectively captured the dynamic changes in grain temperature, achieving higher prediction accuracy than the other three models. Additionally, as shown in Figure 8, the model’s prediction errors were primarily concentrated in the surface layers of the grain pile (9, 3, 4) and (1, 1, 4). The main reason for this phenomenon was that the temperature at the surface of the grain pile was more influenced by meteorological factors. However, the existing models relied solely on temperature data and absolute water potential data from within the grain pile for training, neglecting these external environmental variables. To address this issue, future work will consider incorporating meteorological temperature and humidity as input variables, aiming to further enhance the model’s ability to predict the temperature at the surface of the grain pile by adding external meteorological data.

### 3.3. Potential Risk Early Warning Experiment

To verify the early warning capability of the integrated deep learning framework for grain storage monitoring and risk alert modelling in real grain storage environments, a potential risk warning experiment was designed and conducted. The experimental data were obtained from two distinct granaries: Granary 1, which had experienced mildew risk, and Granary 2, which had undergone condensation risk. The historical grain temperature data, grain moisture data, and the specific occurrence dates and locations of the mildew and condensation events (denoted as T) were collected for 40 days before the event (T − 40 to T − 1) and 4 days after the event (T + 1 to T + 4). These data were used to test the model’s performance in capturing temperature trend changes in the grain storage environment and issuing early warnings for abnormal risks within the grain pile. First, the historical grain temperature data from T − 40 to T − 6 were input into the 3DCNN-LSTM grain temperature prediction model to predict the grain temperature changes for the next 10 days (T − 5 to T + 4). Subsequently, the prediction results were input into the 3D DenseNet-based grain storage state classification model for grain storage state classification, evaluating the status change of the granary. In the experiments with Granary 1 (mildew) and Granary 2 (condensation), the prediction results from the 3DCNN-LSTM model over the 10-day prediction period showed that the MAE for Granary 1 was 0.29 °C and the RMSE was 0.36 °C, while for Granary 2, the MAE was 0.19 °C and the RMSE was 0.22 °C. Compared to the model’s performance on the test set, the prediction errors (MAE and RMSE) for the mildew granary were significantly higher, possibly due to the rapid temperature increase within the grain pile during the mildew event, a temperature change pattern that was not captured in the training data.

**Figure 11:**
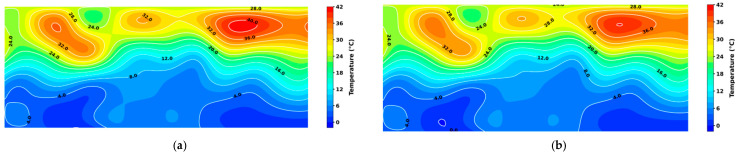

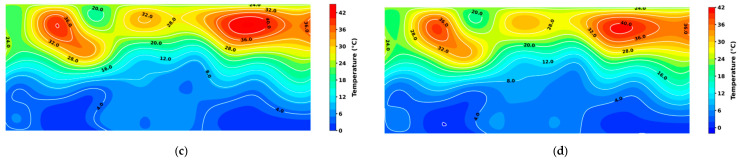
A comparison of the mildew event prediction results by the proposed algorithm with actual temperature field cloud maps. (**a**) Actual temperature field cloud map of the granary on day T; (**b**) predicted temperature field cloud map of the granary on day T; (**c**) actual temperature field cloud map of the granary on day T + 2; and (**d**) predicted temperature field cloud map of the granary on day T + 2.

After inputting the 10-day grain temperature prediction matrix of Granary 1 into the 3D DenseNet-based grain storage state classification model, the system classified the granary state as normal storage from T − 5 to T + 1, and as mildew on T + 2. The T-day grain temperature prediction results were compared with the actual grain temperature values, and the T + 2-day prediction results were compared with the actual grain temperature values, generating a temperature field cloud map for the mildew area of the grain pile. By analyzing the XZ-section temperature field cloud map in Figure 11, comparing Figure 11a,c, it can be observed that the temperature at the surface of the grain pile gradually decreased with the decline in external temperature, while the temperature of the mildew area within the grain pile rose rapidly, and the heated area expanded significantly, verifying the occurrence of mildew. Figure 11b,d show the grain temperature prediction results for the mildew area on T-day and T + 2-day. The algorithm failed to detect mildew when it occurred. By comparing the temperature field cloud maps, it can be seen that the predicted grain temperature in the mildew area was significantly lower than the actual grain temperature (Figure 11b), with the evaluation metrics as a MAE of 0.31 °C and RMSE of 0.47 °C, leading to a 2-day delay in the judgment of mildew risk. The occurrence of this prediction error was likely closely related to the rapid increase in temperature within the grain pile during the mildew event, and the lack of such rapid temperature change patterns in the training data. Nevertheless, the model was still able to issue an early warning 6 days before the mildew event, demonstrating the effectiveness and feasibility of the early warning algorithm.

**Figure 12:**
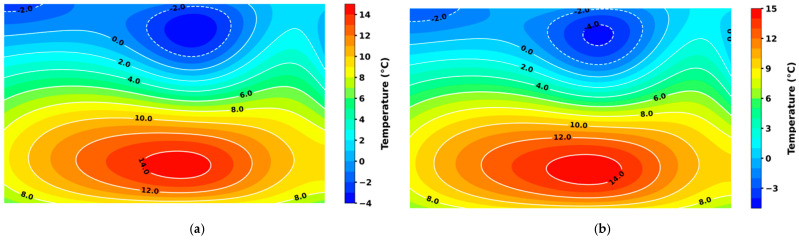

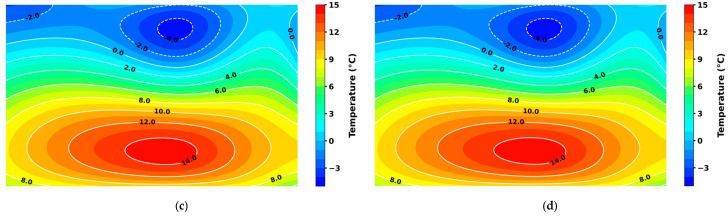
A comparison of the condensation event prediction results from the proposed algorithm with the actual temperature field cloud maps. (**a**) Actual temperature field cloud map of the granary on day T − 1; (**b**) predicted temperature field cloud map of the granary on day T − 1; (**c**) actual temperature field cloud map of the granary on day T; and (**d**) predicted temperature field cloud map of the granary on day T.

In the analysis of condensation risk, the 10-day grain temperature prediction matrix for Granary 1 was input into the 3D DenseNet-based grain storage state classification model. The granary state was classified as normal storage from T − 5 to T − 2, and condensation risk was detected on T − 1. As shown in Figure 12, after prolonged high temperatures in the summer, external heat was gradually conducted into the grain pile, causing the temperature in the central region to rise and a hot core to form. During the late autumn and early winter, the surface temperature of the grain pile dropped rapidly, while heat transfer within the pile remained slow. The central hot core cooled down very gradually, resulting in a significant temperature gradient within the pile. This gradient created a typical “cold skin, hot core” condition, consistent with the characteristics of condensation risk. According to the grain pile multi-field coupling theory, condensation state analysis reveals that when a pronounced temperature gradient exists within the pile, local microflows are formed, with warm air rising and cold air descending [17]. Heat and moisture from the hot core area are carried by these microflows to the cooler surface regions. Heat can dissipate into the external low-temperature environment, while large amounts of water vapor condense upon encountering the cold grain, sometimes even forming condensed moisture, which remains on the surface of the cold grain. By evaluating the prediction results in Figure 12b, it was found that an accurate warning was successfully issued by the model 5 days before the occurrence of condensation risk, with the grain temperature prediction results achieving an MAE of 0.20 °C and an RMSE of 0.23 °C, demonstrating good prediction accuracy.

Through the potential risk warning experiments, the application of the proposed integrated deep learning framework for grain storage monitoring and risk alert in the grain storage environment was validated. Although some prediction delay occurred during the mildew risk warning process, resulting in a 2-day delay in the mildew risk assessment, the model successfully captured the temperature trend in the grain storage environment and issued an effective early warning, demonstrating the potential and effectiveness of this algorithm for grain storage safety monitoring and risk alert. Future work will involve the continued collection of extreme grain temperature variation data related to mildew and condensation events to be incorporated into the training set of the grain temperature prediction model. This will improve the model’s responsiveness in rapidly changing environments and enable more precise predictions of potential mildew and condensation risks within grain piles.

## 4. Conclusions

To address the current deficiencies in grain storage state monitoring we introduced a multi-model fusion-based method for grain storage state monitoring and risk alerting within a deep learning framework. The proposed approach integrates two three-dimensional deep learning models, 3D DenseNet and 3DCNN-LSTM, to effectively process three-dimensional grain temperature data that capture the spatial distribution of temperature fields within grain piles. This integration significantly enhances the understanding of the spatial structure of grain temperature fields, which is essential for real-time monitoring and early risk warning in grain storage. The developed model comprises a 3D DenseNet-based classification module for grain storage states and a 3DCNN-LSTM-based temperature prediction module. The 3D DenseNet module accurately identifies and classifies grain storage states using current grain temperature data, while the 3DCNN-LSTM module predicts dynamic changes in the temperature field within the granary by combining historical grain temperature data with absolute water potential information. The predicted grain temperature data are subsequently fed into the 3D DenseNet model to facilitate early warnings of potential risks, such as condensation and mildew. Experimental results indicate that the 3D DenseNet module exhibits outstanding performance in multi-class classification tasks, achieving an accuracy of 97.38%, precision of 0.9596, recall of 0.9602, and an F1 score of 0.9585, significantly surpassing other models. In temperature prediction tasks, the 3DCNN-LSTM achieved a Mean Absolute Error (MAE) of 0.24 °C and a Root Mean Square Error (RMSE) of 0.28 °C, demonstrating high prediction accuracy. Additionally, the experiments confirmed that utilizing the three-dimensional model and incorporating absolute water potential information substantially improves the accuracy of grain temperature predictions. In assessing the model’s risk warning capabilities, the system successfully captured temperature trends within the grain storage environment and issued effective early warnings for mildew and condensation risks, despite a 2-day prediction delay in mildew risk warnings. These findings validate the feasibility and effectiveness of the proposed model for grain storage safety monitoring and risk alert systems. Future research will focus on further optimizing the model and expanding the dataset, particularly by supplementing it with more extreme temperature change data related to abnormal events, such as mildew and condensation, to improve the model’s responsiveness in rapidly changing environments. Additionally, more environmental factors, such as meteorological temperature and humidity, as well as different types of storage facilities, will be integrated to enhance the model’s comprehensive predictive capabilities in complex, multi-variable environments.

## Figures and Tables

**Table 1:** Classification results of grain storage states in dataset.

Storage Location	Normal Storage	Empty Storage	Aeration	New Grain Addition	Condensation	Mildew
Anhui	76,426	32	10	28	7	1
Fujian	21,682	95	11	0	7	0
Jiangxi	9692	5	35	0	9	10
Henan	6707	34	0	38	15	9
Hubei	12,901	39	27	0	5	3
Hunan	1947	41	0	22	5	0
Guangdong	7468	16	12	0	1	0
Guizhou	8502	39	5	31	11	3
Shanxi	11,430	42	17	3	26	7
Gansu	29,022	51	6	59	14	3
Jilin	13,758	92	86	29	5	0
Xinjiang	21,725	51	103	17	16	9
Total	221,260	537	312	227	121	45

**Table 2:** Statistics of grain temperature data.

	Value
Mean (°C)	7.27
Median (°C)	6.06
Standard Deviation (°C)	11.41
Maximum (°C)	−15.7
Minimum (°C)	38.7
Skewness	0.17
Kurtosis	−0.98

**Table 3:** Hyperparameter value range and optimal settings for 3D DenseNet model.

Parameter	Value Range	Optimal Parameter Settings
Learning Rate	0.0001, 0.001, 0.01	0.001
Base Channels	16, 32, 64	32
Dropout Rate	0.2, 0.3, 0.5	0.3
Num Dense Blocks	3, 4	3
Num Layers Per block	3, 4, 5	4
Compression Rate	0.3, 0.5, 0.7	0.5

**Table 4:** Hyperparameter value range and optimal settings for 3DCNN-LSTM model.

Parameter	Value Range	Optimal Parameter Settings
Learning Rate	0.0001, 0.001, 0.01	0.001
LSTM Hidden Size	64, 128, 256	128
LSTM Layers	2, 3, 4	3
Dropout Rate	0.3, 0.5, 0.7	0.5
Time Window	30, 35, 40	35

**Table 5:** Evaluation coefficients of each model.

Model	Accuracy	Precision	Recall	F1
3D DenseNet	97.38%	0.9596	0.9602	0.9585
3D ResNet	96.62%	0.9506	0.9431	0.9467
3D InceptionV3	94.93%	0.9222	0.9074	0.9135
3D EfficientNet	92.72%	0.8833	0.8752	0.8790

**Table 6:** Comparison of four model evaluation indexes.

Model	MAE (°C)	RMSE (°C)
3DCNN-LSTM	0.24	0.28
3DCNN-GRU	0.29	0.32
LSTM	0.34	0.38
3DCNN-LSTM-1	0.27	0.31

## Data Availability

The data presented in this study are available on request from the corresponding author due to confidentiality restrictions regarding corporate grain storage data.
